# Risk of SARS-CoV-2 infection, severe COVID-19 illness and COVID-19 mortality in people with pre-existing mental disorders: an umbrella review

**DOI:** 10.1186/s12888-023-04641-y

**Published:** 2023-03-20

**Authors:** Federico Bertolini, Anke B Witteveen, Susanne Young, Pim Cuijpers, Jose Luis Ayuso-Mateos, Corrado Barbui, María Cabello, Camilla Cadorin, Naomi Downes, Daniele Franzoi, Michael Elizabeth Gasior, Brandon Gray, Ann John, Maria Melchior, Mark van Ommeren, Christina Palantza, Marianna Purgato, Judith Van der Waerden, Siyuan Wang, Marit Sijbrandij

**Affiliations:** 1 WHO Collaborating Centre for Research and Training in Mental Health and Service Evaluation, Department of Neuroscience, Biomedicine and Movement Sciences, University of Verona, Verona, Italy; 2 Clinical, Neuro and Developmental Psychology, WHO Collaborating Centre for Research and Dissemination of Psychological Interventions, Amsterdam Public Health Institute, VU University, Amsterdam, the Netherlands; 3 International Institute for Psychotherapy, Babeș-Bolyai University, Cluj-Napoca, Romania; 4 Department of Psychiatry, Hospital Universitario de La Princesa, Instituto de Investigación Sanitaria Princesa (IIS-Princesa), Madrid, Spain; 5 Department of Psychiatry, Universidad Autónoma de Madrid, Madrid, Spain; 6 Centro de Investigación Biomédica en Red de Salud Mental, CIBERSAM, Instituto de Salud Carlos III, Madrid, Spain; 7 Sorbonne Université, INSERM, Institut Pierre Louis d’Epidémiologie et de Santé Publique, Social Epidemiology Research Team (ERES), Paris, F75012 France; 8 Population Psychiatry, Suicide and Informatics, Medical School, Swansea University, Swansea, UK; 9 Department of Mental Health and Substance Use, World Health Organization, Geneva, Switzerland

**Keywords:** Umbrella review, Covid-19, Sars-CoV-2, Mental health, Mortality, Pre-existing mental health disorders

## Abstract

**Introduction:**

The COVID-19 pandemic has posed a serious health risk, especially in vulnerable populations. Even before the pandemic, people with mental disorders had worse physical health outcomes compared to the general population. This umbrella review investigated whether having a pre-pandemic mental disorder was associated with worse physical health outcomes due to the COVID-19 pandemic.

**Methods:**

Following a pre-registered protocol available on the Open Science Framework platform, we searched Ovid MEDLINE All, Embase (Ovid), PsycINFO (Ovid), CINAHL, and Web of Science up to the 6th of October 2021 for systematic reviews on the impact of COVID-19 on people with pre-existing mental disorders. The following outcomes were considered: risk of contracting the SARS-CoV-2 infection, risk of severe illness, COVID-19 related mortality risk, risk of long-term physical symptoms after COVID-19. For meta-analyses, we considered adjusted odds ratio (OR) as effect size measure. Screening, data extraction and quality assessment with the AMSTAR 2 tool have been done in parallel and duplicate.

**Results:**

We included five meta-analyses and four narrative reviews. The meta-analyses reported that people with any mental disorder had an increased risk of SARS-CoV-2 infection (OR: 1.71, 95% CI 1.09–2.69), severe illness course (OR from 1.32 to 1.77, 95%CI between 1.19–1.46 and 1.29–2.42, respectively) and COVID-19 related mortality (OR from 1.38 to 1.52, 95%CI between 1.15–1.65 and 1.20–1.93, respectively) as compared to the general population. People with anxiety disorders had an increased risk of SAR-CoV-2 infection, but not increased mortality. People with mood and schizophrenia spectrum disorders had an increased COVID-19 related mortality but without evidence of increased risk of severe COVID-19 illness. Narrative reviews were consistent with findings from the meta-analyses.

**Discussion and conclusions:**

As compared to the general population, there is strong evidence showing that people with pre-existing mental disorders suffered from worse physical health outcomes due to the COVID-19 pandemic and may therefore be considered a risk group similar to people with underlying physical conditions. Factors likely involved include living accommodations with barriers to social distancing, cardiovascular comorbidities, psychotropic medications and difficulties in accessing high-intensity medical care.

**Supplementary Information:**

The online version contains supplementary material available at 10.1186/s12888-023-04641-y.

## Background

The COVID-19 pandemic has affected most of the population worldwide. Disadvantaged population groups are thought to have suffered the most, with possibly among those, people affected by mental disorders. The physical health repercussions have been a particular point of concern, as many known risk factors for severe COVID-19 illness course and COVID-19 related mortality are common in people with mental disorders [1–3]. Firstly, it is unclear whether this population, or specific subgroups, had an increased risk of contracting the SARS-CoV-2 virus than the general population [4, 5]. Shared living accommodations represented contexts in which adhering to social distancing might have been not possible or difficult. Secondly, there has been a focus on the disease course in people with mental disorders. Obesity, diabetes mellitus and cardiovascular disease are common in this population [6, 7]. In addition to that, many psychotropic medications have the potential of a detrimental effect on the respiratory function [8, 9]. Given these premises, it seems highly relevant to assess if and how the COVID-19 pandemic has affected the physical health of people with pre-existing mental disorders. Against this background, an umbrella review was performed aiming to summarize evidence from systematic reviews (SR) and meta-analyses (MAs). This work builds on a wider project of evidence synthesis on the impact of the COVID-19 pandemic on mental health, performed to inform a WHO Scientific Brief [10].

## Methods

### Umbrella review design

We followed published guidelines on performing umbrella reviews [11–13]. An umbrella review consists of a systematic search and assessment of systematic reviews on a specific research question. This allows for the comparison of results of clinical outcomes and provides a clear picture on broad healthcare areas, possibly revealing whether the evidence base is consistent or contradictory. We registered a protocol to define the research methodology to inform the WHO Scientific brief that is publicly available on the Open Science Framework platform [14]. The present work is based on the ‘Question 3 - Are people living with pre COVID-19 existing mental health disorders at (increased) risk of severe illness and mortality and/or of contracting SARS-CoV-2 compared to any other population?’ of the protocol, with the addition of focusing on estimates that have adjusted for confounding.

### Literature search and study selection

An information specialist performed a systematic search on Ovid MEDLINE All, Embase (Ovid), PsycINFO (Ovid), CINAHL, and Web of Science, from December 31, 2019 until October 6, 2021, using a search strategy based on a combination of keywords and text words for reviews, COVID-19, and mental disorders or problems. The search strategy can be found in the supplementary material. This search was designed to inform a wide panel of different research questions on COVID-19 and mental health. We deduplicated search results using Endnote software [15]. Entries were divided into groups, each screened in parallel and independently by two different authors using Rayyan [16]. In case of disagreement, records were conserved. The full text of the remaining articles was assessed against the inclusion and exclusion criteria, again in duplicate and independently. Disagreement was resolved by discussion or with a senior review author.

### Eligibility criteria and data extraction

We considered for inclusion reviews that: (1) were systematic, as defined by having a systematic search on at least one bibliographic database, explicitly reporting primary studies selection criteria and providing a list and synthesis of included studies; (2) considered as primary studies either cohort or cross-sectional or case-control studies; (3) have been published in a peer-reviewed journal; (4) compared people with pre-existing mental disorders (i.e., with a diagnosis of mental disorder established before the pandemic onset) with those without a pre-existing mental disorder; and (5) assessed at least one of the following outcomes: (a) risk of contracting SARS-Cov-2 infection, (b) risk of severe COVID-19 illness, (c) risk of long-term physical symptoms after COVID-19 illness, (d) mortality from SARS-CoV-2 infection. For the outcome ‘risk of severe illness’, we considered definitions as provided by original systematic reviewers, or proxy outcomes such as hospitalization. We did not exclude reviews on the basis of language of publication. In the case of reviews considering several pandemics, we planned to include only those that reported separated data for the COVID-19 pandemic. We considered systematic reviews independently of whether a meta-analysis was performed. Two authors independently and in parallel extracted the following data: author, date of publication, timeframe covered by the search, population and comparator, design of included studies, inclusion and exclusion criteria, outcomes, risk of bias assessment strategy, how exposure to SARS-CoV-2 was established, number of included studies and participants, and outcomes of interest with I^2^ statistic as a measure of heterogeneity. In terms of meta-analysis, we aimed to summarize effect size in terms of adjusted Odds Ratio (aOR), if that was not available, we considered the most adjusted model available.

### Assessment of quality

Two independent authors assessed the quality of the included systematic reviews using the AMSTAR 2 tool [17]. For systematic reviews that did not perform a meta-analysis, items 11, 12 and 15 were not considered. Also, small adaptations to some items were performed to make them more suitable to score (see the supplementary material). For an overall rating, we used the proposed scheme by Shea and colleagues that provides judgments of high, moderate, low or critically low confidence [17].

## Results

### Selection and inclusion of systematic reviews

**Fig. 1:**
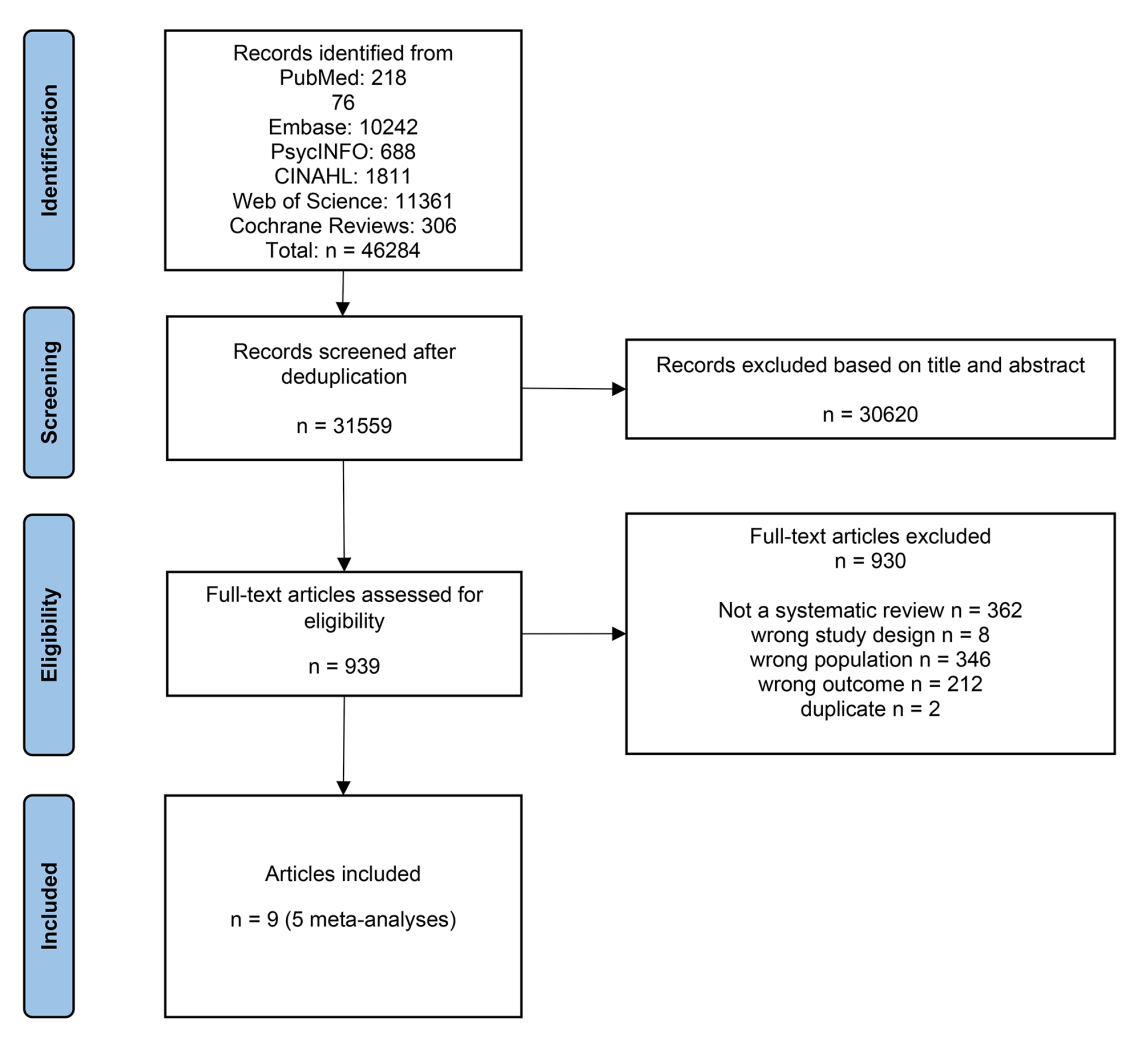
PRISMA flowchart of studies selection

The systematic search provided 46,284 record entries, reduced to 31,559 after deduplication. 939 records were selected for full text retrieval by screening titles and abstracts. Of these, nine were finally included after comparing full texts against inclusion and exclusion criteria. Figure 1 reports the details of the study selection process.

### Characteristics of included systematic reviews

Of the nine included reviews, five performed a meta-analysis on at least one of the outcomes of interest [18–22], while the remaining four were of a narrative nature [23–26]. Table 1 summarises the characteristics of the included reviews.

**Table 1:** Characteristics of included systematic reviews

Author, year	Design	Timeframe covered by bibliographic search	Design of included studies	Population / comparator	Countries	Effect size estimation methods	Number of included Studies	Outcomes
Ceban 2021	Systematic review with meta-analysis	From inception to February 1, 2021	Case-control and cohort studies	People with a mood disorder / people without mood disorders	USA, South Korea, Spain, Italy, Turkey, UK, Israel	Random effect model pooling adjusted and crude ORs. Preference given to adjusted ORs when available. Subgroups analyses according to adjustment status	21 (15 aOR, 6 crude ORs)	1- Susceptibility (laboratory testing, ICD-10, EHR, and/or clinical judgment) 2- Hospitalization 3- Occurrence of severe events (includes oxygen therapy) 4- Death
Fond 2021	Systematic review with meta-analysis	From inception to February 12, 2021	Population-based cohort studies based on medico-administrative health databases or a health care data warehouse	People with any mental disorder (includes substance abuse disorders and ADHD) / people without a mental disorder	Denmark, France, Israel, South Korea, Spain, UK, USA	Random effects models, separate pooling of aORs and crude ORs	21	1- Mortality 2- ICU admission
Fornaro 2021	Scoping review (no meta-analysis)	From inception up to April 24, 2021	Cross-sectional, prospective cohort, retrospective cohort studies, case reports/series	People with Bipolar disorder / NA	Italy, UK, Australia, USA, the Netherlands, India, China		14	Identified themes: 1- Impact of COVID-19-related stressors on BD 2- Impact of COVID-19 on mental health service utilization among people with BD 3- Impact of BD on the risk of acquiring SARS-cov-2 infection 4- Engagement in preventative behaviors among people with BD
Karaoulanis 2021	Systematic review without meta-analysis	From inception up to 21 March 2021	Unreported	People with Schizophrenia / people without Schizophrenia	USA, Korea, France, Israel		7	1- Susceptibility (being infected) 2- Mortality
Lemieux 2020	Rapid review	From December 2019 up 18 August 2020	Opinion pieces by experts (editorials, letters to the editor, position papers and other correspondences), rapid literature reviews, narrative (i.e., Non-systematic) literature reviews, empirical studies (surveys and descriptive/case studies)	People with mental illness in secure settings / NA	USA, China, Italy, UK, France, Germany, Ireland, Singapore, Brazil, Canada, Spain, Australia, India, Israel, Poland, New Zealand, South Korea, Scandinavia, Switzerland		69	Narrative review on strategies, challenges and recommendations for dealing with the COVID-19 outbreak in secure settings for persons with mental illness
Liu 2021	Systematic review with meta-analysis	From inception to January 16, 2021	Cohort, case-control, cross-sectional studies and case series	People with mental disorders (includes sleep disorders and ADHD) / people without mental disorders	USA, Italy, Korea, UK, China, Iran, Brazil, Spain, Turkey, Israel, Switzerland, the Netherlands, Denmark, France, Belgium, Sweden, Russia, Peru, Germany, Malaysia, Poland	Random effect models pooling adjusted and crude ORs. Subgroups analyses according to adjustment status for ‘any mental disorder’ population only	149	1- Susceptibility (positive laboratory results, diagnosis in conjunction with clinical presentation) 2- Illness severity (hospitalization, ICU admission, or requirement for other special treatment (e.g., oxygen therapy, mechanical ventilator, extracorporeal membrane oxygenation, and cardiopulmonary resuscitation)) 3- Death
Murphy 2021	Scoping review	From 1 January to 31 December 2020	Primary research papers including qualitative, quantitative and mixed methods study designs	People with mental disorders / NA	Italy, USA, China, Canada, Germany, Spain, UK, Ireland, Australia, India, Switzerland, France, Iran, Turkey, Poland, Pakistan, Bosnia and Herzegovina		30	Narrative review of factors influencing health outcomes in people with pre-existing mental health conditions during the pandemic; impact of the pandemic on the health of people with pre-existing mental health conditions; Strategies or measure to support people with pre-existing mental health conditions during the pandemic
Toubasi 2021	Systematic review with meta-analysis	From inception to 15 February 2021	Cohort, case control	People with mental disorders / people without mental disorders	UK, Japan, South Korea, France, Scotland, Israel, USA	Random effects models, pooling both adjusted and crude ORs. Sensitivity analysis including aOR only	16 (aOR: 5)	1- Death or Severe illness (ICU admission and ventilation)
Vai 2021	Systematic review with meta-analysis	From 1 January 2020 to 5 March 2021	Cross-sectional, longitudinal studies	People with mental disorders (includes substance use disorders and intellectual disabilities)/ people without mental disorders	UK, Japan, South Korea, France, Scotland, Sweden, USA	Random effects models pooling crude ORs. Sensitivity analysis polling aORs only.	23	1- Death 2- Hospitalization 3- ICU admission

aORs: adjusted Odds Ratios, ADHD: Attention-deficit hyperactivity disorder, BP: Bipolar disorder, ICU: intensive care unit, NA: Not applicable, ORs: Odds Ratios, UK: United Kingdom, USA: United States of America,

Systematic reviews with meta-analyses (MAs) generally had a search covering up to the first quarter of 2021. They included primary studies with mainly a cohort design, with the notable exceptions of the review by Liu and colleagues which considered case-series too and the review by Fond and colleagues which considered population-based cohort studies. MAs included a mean of 45 studies and a median of 21, the review by Liu and colleagues being an outlier with 149 included studies. Most of the MAs considered people with any mental disorder, possibly providing subgroups for specific disorders groups, while the review by Ceban and colleagues only focused on people with a mood disorder. Included studies covered a wide range of countries but with a general lack of representation from low- and middle-income countries. Infection by SARS-CoV-2 was defined by laboratory testing, ICD-10, electronic health records, or clinical judgment in the review by Ceban and colleagues and by laboratory results and diagnosis in conjunction with clinical presentation in the review by Liu and colleagues. Severe illness was defined as ICU admission, mechanical ventilatory support, oxygen therapy, extracorporeal membrane oxygenation, acute respiratory distress syndrome, and/or cardiopulmonary resuscitation by Ceban and colleagues and as hospitalization, ICU admission, or requirement for other special treatment (including oxygen therapy) by Liu and colleagues. For the review by Vai and colleagues, which does not have a “severe illness” outcome, we considered the outcome ‘hospitalisation’. For the review by Toubasi and colleagues, we considered the pooled mortality and severe illness outcome, as a separate estimate for mortality only was not available. Notably, no review was available to inform on the long-term physical symptoms after SARS-CoV2 infection. Four reviews reported estimates based on pooling adjusted ORs only [18, 19, 21, 22]. The review by Vai and colleagues reported a fully adjusted model only when considering people with any mental disorder, for the diagnostic groups we have then considered the ‘partially adjusted model’, where review authors considered aORs pooled together with crude ORs when an adjusted figure was not available from primary studies.

The four narrative reviews varied in their specific design, with two scoping reviews [25, 26], one rapid review [23], and one systematic review without meta-analysis [24]. They considered a wide range of study designs with the review by Lemieux and colleagues considering opinion pieces and other reviews; as for population of interest, they considered people with bipolar disorder [25], people with schizophrenia [24], people with mental illness in secure settings [23], and generally people with mental disorders [26].

### Quality of included reviews

The AMSTAR 2 rated level of quality for all the MAs was “low”, with the exception of the review by Liu and colleagues with “high” and the review by Fond and colleagues with “critically low”. The review by Liu and colleagues did not have any weaknesses in critical items, while all other MAs did not report the list of excluded studies with reasons for exclusion; the review by Fond and colleagues also did not account for the impact of risk of bias in primary studies on the results. The level of quality for the narrative reviews was “critically low” with the exception of the review by Fornaro and colleagues (“low”), mainly because of a lack of risk of bias assessment and protocol registration. See the supplementary material for detailed AMSTAR 2 evaluation of the included reviews.

### Risk of SARS-CoV-2 infection

**Fig. 2:**
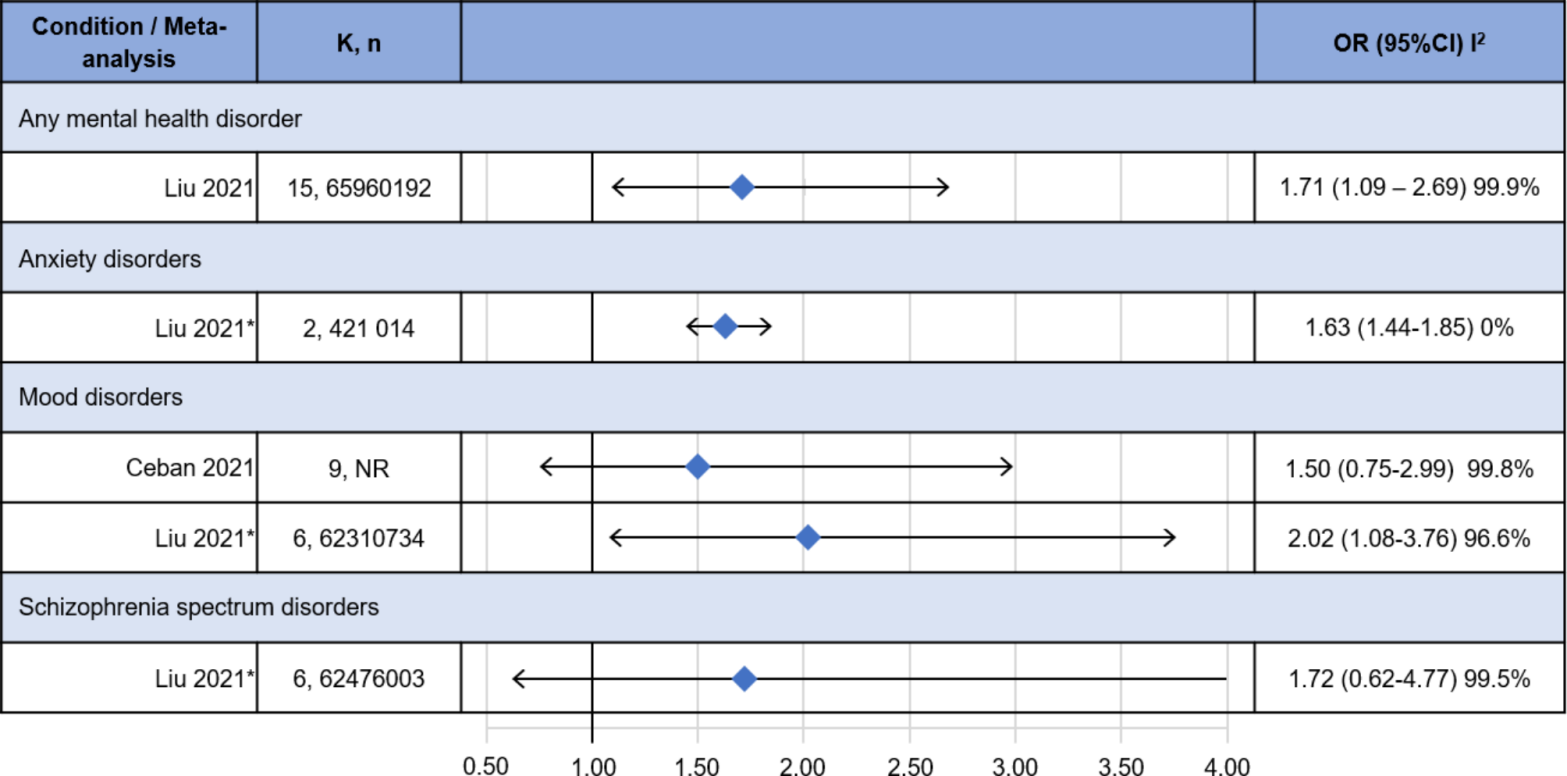
Risk of contracting SARS-CoV-2 infection. CI: confidence interval; K: number of included studies; OR: Odds ratio; n: total number of included participants; NR: not reported; *: partially adjusted model

Two MAs informed on the association between Sars-CoV-2 infection and having a pre-existing mental disorder compared to not having a pre-existing mental disorder (Fig. 2) [18, 19].

For people with any mental disorder, Liu and colleagues found a statistically significant positive association (OR: 1.71, 95% CI 1.09–2.69). For people with anxiety disorders, Liu 2021 and colleagues in a partially adjusted model found a statistically significant positive association although this effect size relies on two studies only (OR 1.63, 95%CI 1.44–1.85). For people with mood disorders, Liu 2021 and colleagues in a partially adjusted model found a statistically significant positive association (OR: 2.02, 95%CI 1.08–3.76), while Ceban and colleagues did not find a statistically significant association (OR: 1.50, 95%CI 0.75–2.99). For people with schizophrenia spectrum disorders, Liu and colleagues, in a partially adjusted model, did not find a statistically significant association (OR: 1.72, 95%CI 0.62–4.77). The level of statistical heterogeneity was been generally very high, with most I^2^ statistics over 95%, with the exception of the estimate for people with mood disorders by Liu and colleagues (0%). I^2^ was not reported in Ceban et al., 2021.

### Risk of severe illness

**Fig. 3:**
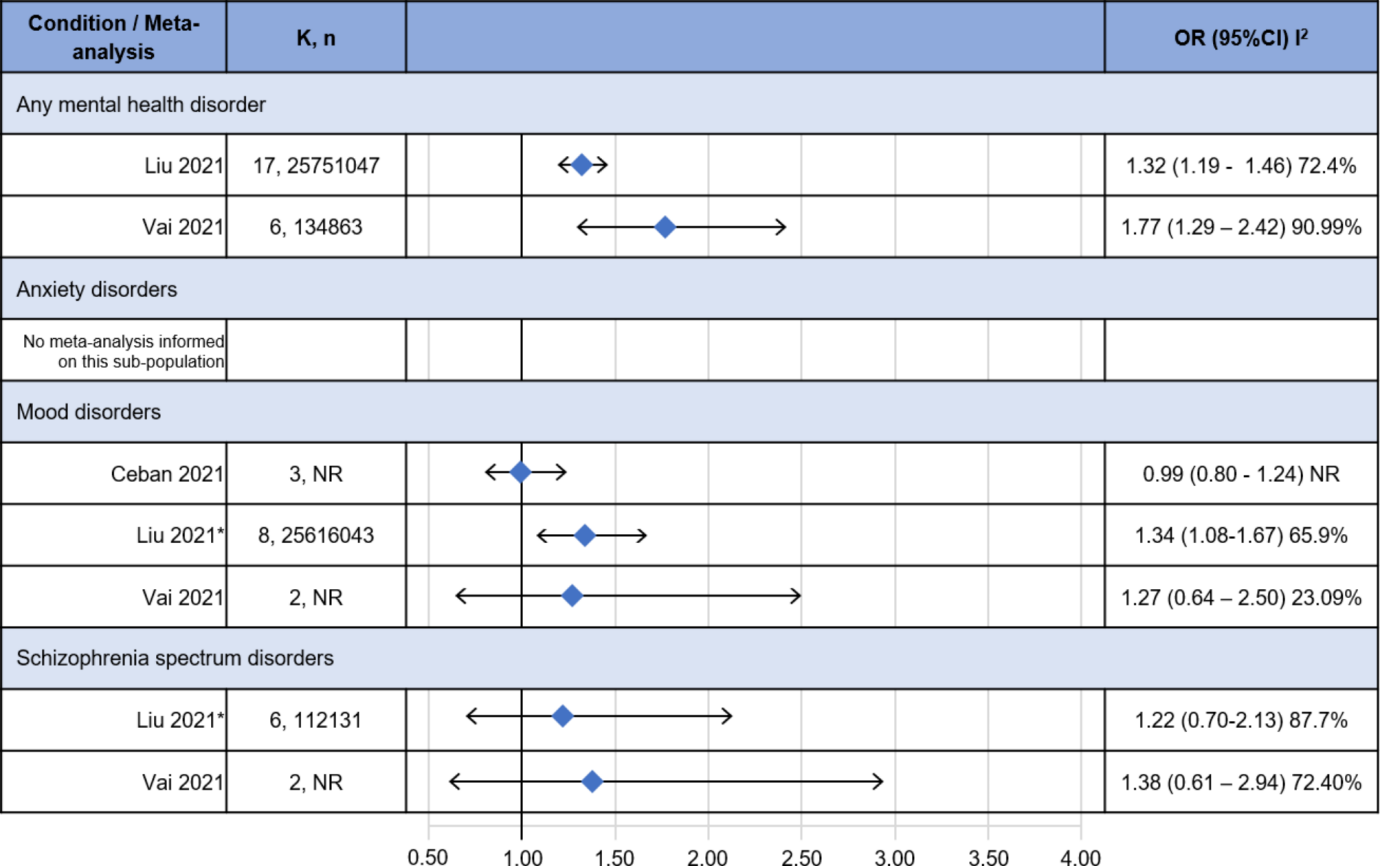
Risk of severe COVID-19 illness. CI: confidence interval; K: number of included studies; OR: Odds ratio; n: total number of included participants; NR: not reported; *: partially adjusted model

Three MAs informed on the association between a severe course of COVID-19 and having a pre-existing mental disorder compared to not having a pre-existing mental disorder (Fig. 3) [18–20].

For people with any mental disorder, both the review by Liu and colleagues and Vai and colleagues found a statistically significant positive association (OR: 1.32, 95%CI 1.19–1.46 and OR: 1.77, 95%CI 1.29–2.42, respectively). No study informed on people with anxiety disorders. For people with a mood disorder, Ceban and colleagues found no association (OR: 0.99, 95%CI: 0.80–1.24), Liu and colleagues in a partially adjusted model found a statistically significant positive association (OR: 1.34, 95%CI 1.08–1.67) while Vai and colleagues did not find a statistically significant association (OR 1.27, 95%CI 0.64–2.50). For people with a schizophrenia spectrum disorder, both the reviews by Liu and colleagues and Vai and colleagues did not find a statistically significant association (OR: 1.22, 95%CI 0.70–2.13 and OR: 1.38, 95%CI 0.61–2.94, respectively). The level of statistical heterogeneity was moderate to very high, with an I^2^ statistic between 65 and 100%, but low for the estimate for people with mood disorders by Vai and colleagues (23%). I^2^ was not reported in Ceban et al., 2021.

### COVID-19 related mortality

**Fig. 4:**
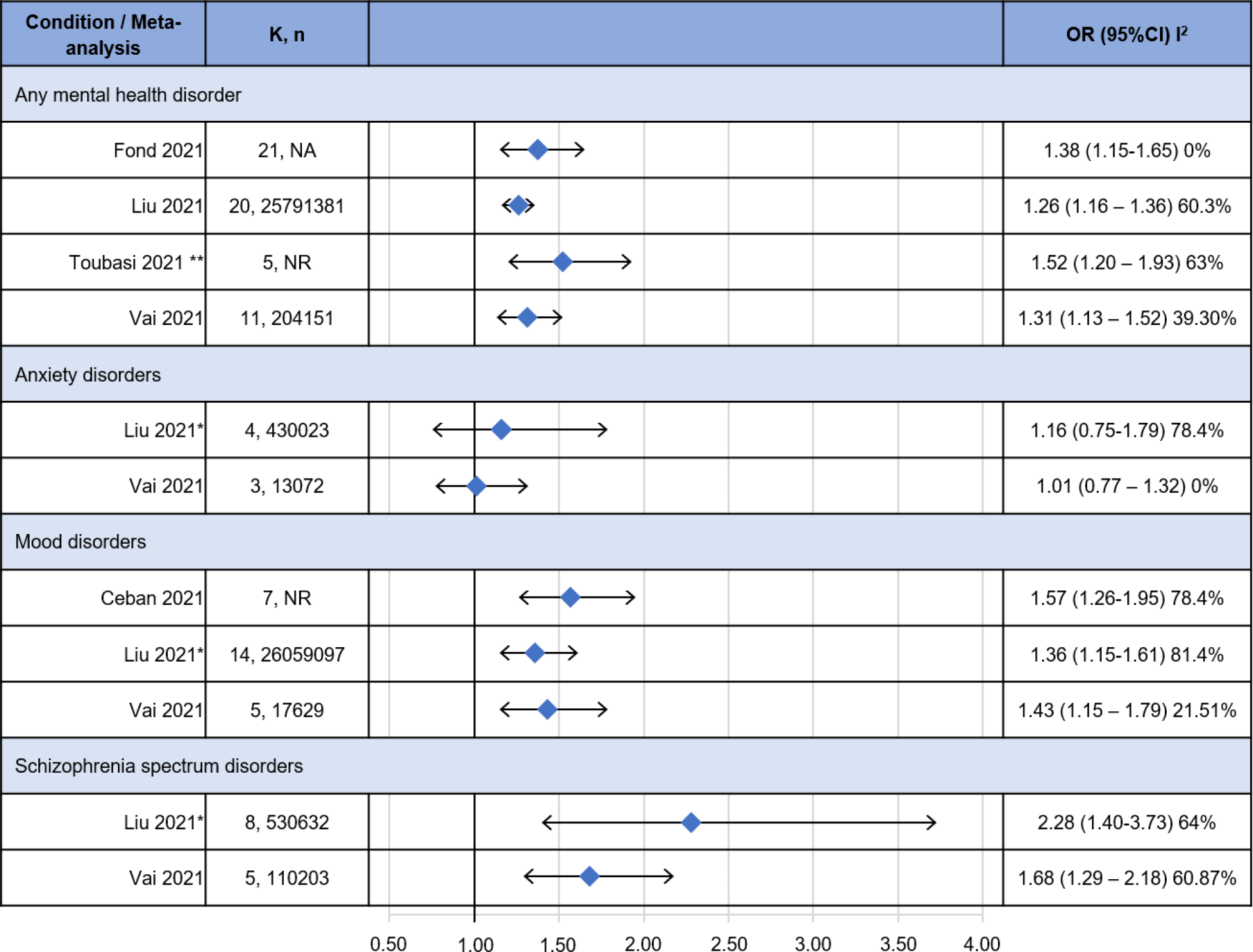
COVID-19 related mortality risk. CI: confidence interval; K: number of included studies; OR: Odds ratio; n: total number of included participants; NR: not reported; *: partially adjusted model; **: considers mortality and severe illness together

Four MAs informed on the association between mortality and having a pre-existing mental disorder compared to not having a pre-existing mental disorder (Fig. 4) [19–22].

For people with any mental disorder all four MAs found a statistically significant positive association, ranging from an OR of 1.38 (95%CI: 1.15–1.65) for the review by Fond and colleagues to 1.52 (95%CI: 1.20–1.93) for the review by Toubasi and colleagues (which however considered in this outcome severe illness cases as well). For people with anxiety disorders both the reviews by Liu and colleagues, in a partially adjusted model, and by Vai and colleagues did not find a statistically significant association (OR: 1.16, 95%CI 0.75–1.79 and OR: 1.01, 95%CI 0.77–1.32, respectively). For people with mood disorders, all three informing MAs found a statistically significant positive association with ORs ranging from 1.36 (95%CI: 1.15–1.79, Liu and colleagues, partially adjusted model) to 1.57 (95%CI: 1.26–1.95 Vai and colleagues). For people with schizophrenia spectrum disorders both the reviews by Liu and colleagues, in a partially adjusted model, and by Vai and colleagues found a positive association (OR 2.28, 95%CI 1.40–3.73, and OR 1.68, 95%CI 1.29–2.18, respectively). The level of statistical heterogeneity has been generally moderate to considerable, with an I^2^ statistic between 60% and 81.4%, but for the review by Vai and colleagues for the estimate for people with any mental disorder (39%), and low for the estimate for people with anxiety disorders (0%) and mood disorders (22%). The I^2^ has not been reported in Ceban et al., 2021.

### Narrative reviews

The narrative reviews corroborated meta-analytic findings by indicating that patients with serious pre-COVID-19 mental disorders show adverse health outcomes related to COVID-19 infection in terms of higher severity and mortality. Fornaro and colleagues [25] performed a scoping review on clinical and public health themes for people with bipolar disorder. They identified four major themes from the 14 included papers, among which was the impact of having bipolar disorder on the risk of contracting the SARS-CoV-2 infection. For this theme, one study reported an increased risk of infection contraction for people with bipolar disorder [27]. This study was considered by the MAs previously reported. Karaoulanis and Christodoulou performed a systematic review without meta-analysis on infection rates and mortality in people with schizophrenia spectrum disorders. The included studies suggest an increased infection and mortality risk; these studies have been included in the MAs previously reported. Lemieux and colleagues performed a rapid review on the management of COVID-19 for people with mental illness in secure settings. They considered a wide range of publications including opinion pieces and other reviews and report greater morbidity and mortality. Murphy and colleagues conducted a scoping review on the impact of COVID-19 and related restrictions on people with pre-existing mental disorders. They reported an increased infection risk in this population.

## Discussion

To our knowledge this is the first umbrella review aimed at summarizing evidence on the impact of the COVID-19 pandemic on health outcomes in people with pre-existing mental disorders. Compared to the individual systematic reviews previously published, this umbrella review contextualizes the single piece of evidence, and provides an overview for different mental disorders, while systematic reviews have so far focused on specific diagnostic groups or considered only some of the outcomes of interest on the physical health repercussions of people with mental disorders. Two reviews considered several diagnostic groups and outcomes, however one employed only partially adjusted models within diagnostic groups [19], and the other could include a considerably smaller number of studies [20]. Overall, we found consistent results across the various reviews. Of interest, we found no previous reviews exploring the long-term effects of SARS-CoV-2 infection.

Having any mental disorder was found to be associated with a higher likelihood of contracting the SARS-CoV-2 infection, a more severe COVID-19 illness, and higher mortality. We found different risks for different disorder groups. People with pre-existing anxiety disorders had an increased risk of contracting the SARS-CoV-2 infection; for people with mood disorders there was conflicting evidence of increased risk for severe COVID-19 course, and evidence of increased mortality; people with schizophrenia spectrum disorders had an increased risk of mortality, but there was no clear evidence of increased severe illness course for people with schizophrenia spectrum disorders. Notably, we found no review assessing the association between long-term physical symptoms after COVID-19 and having a pre-existing mental disorder.

Having any mental disorder has been associated with an increased risk of contracting the SARS-CoV-2 infection. Looking at the data for the specific diagnostic groups, however, we observe that the association is confirmed for people with an anxiety disorder (and in an estimate based on only two studies) only, while for people with mood disorders the evidence is not consistent across reviews, and for people with a schizophrenia spectrum disorder the association is not statistically significant with a very wide confidence interval, making it hard to draw clear conclusions. Many people with a mental disorder live in shared households, nursing homes, therapeutic communities, or are inpatients. It has been noted that such settings pose challenges in putting into practice infection control measures [28]. In addition to that, people in an acute phase of a mental disorder might find it difficult to understand the need for and adhere to behavioural means of social distancing [29]. Still, these considerations regard mostly people with serious mental illness such as bipolar disorder or schizophrenia, adding difficulty to the interpretation of these results.

Having a mental disorder was associated with having a more severe illness course and increased mortality. This could be partially explained by the increased risk of contracting the SARS-CoV-2 infection, but as the three disorder groups showed differential patterns, we argue that additional factors may have influenced this outcome. In particular, anxiety disorders were not associated with increased mortality despite an increased infection risk, and indeed the results are compatible with a random distribution in terms of effect sizes and confidence intervals. Mood disorders and schizophrenia spectrum disorders have been associated with increased mortality from COVID-19 without conclusive evidence of increased infection risk. Several factors might come into play in determining such a negative outcome. People with severe mental illness, such as bipolar disorder and schizophrenia, more frequently present with high BMI, diabetes mellitus, generally limited exercise tolerance, and are more likely to also smoke and have substance abuse disorders [6, 7, 30]. All of these are known risk factors for severe COVID-19 and for COVID-19 related mortality [2, 3, 31]. Although the use of adjusted odds ratios should have mitigated the impact of these risk factors in the estimate of effect sizes there was high heterogeneity in terms of adjusted factors used by primary studies. Therefore, it is possible that some factors, such as BMI, might not have been appropriately accounted for [32]. Many people with these disorders would have received an antipsychotic (and potentially benzodiazepines), with a possible negative impact on respiratory function [8]. However, there is limited evidence to support these hypotheses and future research is needed to fill this gap in knowledge. Moreover, we should take into consideration that socio-economic factors and stigma might have influenced the access to medical care of these persons [33]. Many primary studies were conducted during the initial phases of the pandemic when medical resources were scarce compared to needs, access to intensive care units was limited and subject to stringent triage, and no vaccine was available yet. The finding that for people with schizophrenia spectrum disorders there was no increased risk for a more severe disease course, but higher risk for COVID-19 related mortality, is somewhat puzzling. The reviews have used slightly different definitions of “severe illness”, but all considered it as an operational composite outcome where many different events qualified, including oxygen therapy. Oxygen therapy has been a widespread need in COVID-19 patients, as arterial hypoxemia is a major feature of the disease [34]. It is possible that the definition of severe illness was therefore excessively sensitive and did not allow for the identification of differences between people with and without a pre-existing mental disorder. Another possible explanation is that people with schizophrenia might have been disproportionately affected by sudden death events. We know that cardiac sudden death events have been shown to be associated with COVID-19 [35] and that people with schizophrenia have a higher frequency of cardiovascular disease which may predispose to such events [36]. However, there is no direct evidence, and the use of adjusted estimates should have compensated for the increased cardiovascular burden. Overall, the mismatch between the risk of severe disease and mortality reinforces the need to better investigate factors associated with the increased mortality risk.

The findings of this umbrella review should be put into the context of some limitations. For the various diagnostic groups, the number of included primary studies has been limited, especially for anxiety disorders. Mood disorders include both depression and bipolar disorder, two disorders with different pharmacological approaches and neuro-inflammatory profiles. Additionally, information on the disease status of participants (remission or relapse) has generally not been considered. Low- and middle-income countries have been scarcely represented in the primary studies included by the reviews. The publication timeframe covered by the meta-analyses span to the first quarter of 2021; as such, these findings depict the first year of the pandemic. The general landscape has since changed, thanks to health care systems adaptations, improved COVID-19 treatments and importantly, the introduction of vaccines. There is, however, conflicting evidence regarding vaccine uptake rates in people with mental disorders, possibly due to regional differences [37, 38]. The reviews considered different study designs for inclusion; notably, Liu and colleagues considered case series. The review by Vai and colleagues informed on many diagnostic groups, but in only partially adjusted models. There has been considerable heterogeneity across all outcomes, which the meta-analyses struggled to explain. Heterogeneity likely reflects methodological and qualitative differences among the primary studies. Moreover, this high heterogeneity and the generally wide confidence intervals limit the accuracy of the estimates.

In light of these findings, there are relevant questions for future research. The new Omicron variant of the SARS-CoV-2 virus spreads more easily but usually causes less severe illness [39]. Assessing if this holds true for people with a pre-exiting mental disorder as a risk factor would be valuable. In parallel, there is still limited evidence on vaccine hesitancy and uptake rate in this population. Moreover, the topic of long-term physical symptoms after COVID-19 in people with mental disorders remains scarcely investigated. Addressing these three points would allow for more effective health care planning and possibly targeted intervention to address vaccine hesitancy.

## Conclusion

The COVID-19 pandemic has affected people with pre-existing mental disorders more severely than people without in terms of physical health. People with pre-existing mental disorders, and especially those with mood or schizophrenia spectrum disorders, should have been considered at risk of severe course and increased mortality from COVID-19, similar to other identified risk groups such as patients with somatic health conditions.

## Electronic supplementary material

Below is the link to the electronic supplementary material.

Supplementary Material 1

## Data Availability

all data generated or analysed during this study are included in this published article and its supplementary information file and the original studies’ publications.

## Abbreviations

aOR: Adjusted Odds Ratio
ICU: Intensive Care Unit
MAs: Meta-Analyses
OR: Odds Ratio
SR: Systematic Review
