# Supplementary material for: Risk of SARS-CoV-2 infection, severe COVID-19 illness and COVID-19 mortality in people with pre-existing mental disorders: an umbrella review
Source: BMC Psychiatry. 2023 Mar 20;23:181. doi: 10.1186/s12888-023-04641-y (PMC10026202; doi:10.1186/s12888-023-04641-y)
Supplement: Supplementary file 1 — Supplementary Material 1 [file 12888_2023_4641_MOESM1_ESM.docx]

**Risk of SARS-CoV-2 infection, severe COVID-19 illness and COVID-19 mortality in people with pre-existing mental disorders: an umbrella review**

**Supplementary material**

**Search strategies for each database**:

Note: All searches were restricted to time-period from December 31, 2019 onwards (filter) and performed at October 6, 2021

EMBASE

(('coronavirus'/exp OR 'corona virus'/exp OR 'coronavirus infect*' OR 'corona virus infect*' OR 'betacoronavirus*' OR 'beta coronavirus*' OR 'beta corona virus*') AND or wuhan or hubei or AND 'severe acute respiratory syndrome coronavirus 2'/exp OR 'sars cov-2'/exp OR 'sarscov 2' OR 'sarscov2' OR 'cov2' OR 'sars 2' OR 'covid'/exp OR 'coronavirus 2' OR 'covid19'/exp OR 'covid-19'/exp OR 'covid 19 or ncov' OR 'new corona virus' OR 'new coronavirus' OR 'novel coronavirus' OR 'novel corona virus or ncp or corona' OR 'pandemic'/exp OR 'global pandemic' OR 'epidemic'/exp OR 'global epidemic or coronavirinae') AND ('mental or psycholog*' OR 'distress'/exp OR psychiatr* OR 'social stigma'/exp OR 'stigma'/exp OR 'fear'/exp OR 'phobia'/exp OR 'anxiety or anxi*' OR 'stress'/exp OR 'worry'/exp OR 'physiological stress'/exp OR angst OR depress* OR 'mood or irritab*' OR sad* OR affect* OR 'fatigue'/exp OR hope* OR neurotic* OR 'grief or griev* or bereav*' OR 'loss'/exp OR 'burnout'/exp OR 'burned out' OR 'burnt out' OR 'trauma'/exp OR trauma* OR 'post-traumatic' OR 'post-trauma' OR 'ptsd'/exp OR 'post traumatic stress disorder'/exp OR 'post-traumatic stress disorder'/exp OR 'posttraumatic stress disorder'/exp OR 'anger'/exp OR 'substance abuse'/exp OR 'substance use'/exp OR 'substance use disorder'/exp OR 'substance dependence'/exp OR 'drug use'/exp OR 'drug abuse'/exp OR 'drug dependence'/exp) AND (review:ab,ti OR 'systematic review':ab,ti OR 'narrative review':ab,ti OR 'meta-analysis':ab,ti OR 'metaanalysis':ab,ti OR 'meta analysis':ab,ti OR 'literature review or synthes*':ab,ti)

MEDLINE

((coronavirus OR "corona virus" OR "coronavirus infect*" OR "corona virus infect*" OR "betacoronavirus*" OR "beta coronavirus*" OR "beta corona virus*" OR wuhan OR hubei OR "severe acute respiratory syndrome coronavirus 2" OR "SARS CoV-2" OR "SARSCoV 2" OR "SARSCoV2" OR "cov2" or "sars 2" OR COVID OR "coronavirus 2" OR "covid19" OR "Covid-19" OR "Covid 19" OR nCov OR "new corona virus" OR "new coronavirus" OR "novel coronavirus" OR "novel corona virus" OR ncp OR corona OR pandemic OR "global pandemic" OR epidemic OR "global epidemic" OR coronavirinae)

AND (mental OR psycholog* OR "distress" OR "psychiatr*" OR "social stigma" OR stigma OR fear OR phobia OR anxiety OR anxi* OR stress OR worry OR "physiological stress" OR angst OR depress* OR mood OR irritab* OR sad* OR affect* OR fatigue OR hope* OR neurotic* OR grief OR griev* OR bereav* OR loss OR burnout OR "burned out" OR "burnt out" OR trauma OR trauma* OR post-traumatic OR post-trauma OR PTSD OR "Post traumatic stress disorder" OR "post-traumatic stress disorder" OR "posttraumatic stress disorder" OR anger OR "substance abuse" OR "substance use" OR "substance use disorder" OR "substance dependence" OR "drug use" OR "drug abuse" OR "drug dependence"))

AND (review[Title/Abstract] OR "systematic review"[Title/Abstract] OR "narrative review"[Title/Abstract] OR "meta-analysis"[Title/Abstract] OR "metaanalysis"[Title/Abstract] OR "meta analysis"[Title/Abstract] OR "literature review"[Title/Abstract] OR synthes*[Title/Abstract])

PsycInfo

TX ( coronavirus OR "corona virus" OR "coronavirus infect*" OR "corona virus infect*" OR "betacoronavirus*" OR "beta coronavirus*" OR "beta corona virus*" OR wuhan OR hubei OR "severe acute respiratory syndrome coronavirus 2" OR "SARS CoV-2" OR "SARSCoV 2" OR "SARSCoV2" OR "cov2" or "sars 2" OR COVID OR "coronavirus 2" OR "covid19" OR "Covid-19" OR "Covid 19" OR nCov OR "new corona virus" OR "new coronavirus" OR "novel coronavirus" OR "novel corona virus" OR ncp OR corona OR pandemic OR "global pandemic" OR epidemic OR "global epidemic" OR coronavirinae ) AND TX ( mental OR psycholog* OR "distress" OR "psychiatr*" OR "social stigma" OR stigma OR fear OR phobia OR anxiety OR anxi* OR stress OR worry OR "physiological stress" OR angst OR depress* OR mood OR irritab* OR sad* OR affect* OR fatigue OR hope* OR neurotic* OR grief OR griev* OR bereav* OR loss OR burnout OR "burned out" OR "burnt out" OR trauma OR trauma* OR post-traumatic OR post-trauma OR PTSD OR "Post traumatic stress disorder" OR "post-traumatic stress disorder" OR "posttraumatic stress disorder" OR anger OR "substance abuse" OR "substance use" OR "substance use disorder" OR "substance dependence" OR "drug use" OR "drug abuse" OR "drug dependence" ) AND AB ( review OR "systematic review" OR "narrative review" OR "meta-analysis" OR "metaanalysis" OR "meta analysis" OR "literature review" OR synthes* )

CINAHL

TX ( TX ( coronavirus OR "corona virus" OR "coronavirus infect*" OR "corona virus infect*" OR "betacoronavirus*" OR "beta coronavirus*" OR "beta corona virus*" OR wuhan OR hubei OR "severe acute respiratory syndrome coronavirus 2" OR "SARS CoV-2" OR "SARSCoV 2" OR "SARSCoV2" OR "cov2" or "sars 2" OR COVID OR "coronavirus 2" OR "covid19" OR "Covid-19" OR "Covid 19" OR nCov OR "new corona virus" OR "new coronavirus" OR "novel coronavirus" OR "novel corona virus" OR ncp OR corona OR pandemic OR "global pandemic" OR epidemic OR "global epidemic" OR coronavirinae ) AND TX ( mental OR psycholog* OR "distress" OR "psychiatr*" OR "social stigma" OR stigma OR fear OR phobia OR anxiety OR anxi* OR stress OR worry OR "physiological stress" OR angst OR depress* OR mood OR irritab* OR sad* OR affect* OR fatigue OR hope* OR neurotic* OR grief OR griev* OR bereav* OR loss OR burnout OR "burned out" OR "burnt out" OR trauma OR trauma* OR post-traumatic OR post-trauma OR PTSD OR "Post traumatic stress disorder" OR "post-traumatic stress disorder" OR "posttraumatic stress disorder" OR anger OR "substance abuse" OR "substance use" OR "substance use disorder" OR "substance dependence" OR "drug use" OR "drug abuse" OR "drug dependence" ) AND AB ( review OR "systematic review" OR "narrative review" OR "meta-analysis" OR "metaanalysis" OR "meta analysis" OR "literature review" OR synthes* ) ) AND TX ( mental OR psycholog* OR "distress" OR "psychiatr*" OR "social stigma" OR stigma OR fear OR phobia OR anxiety OR anxi* OR stress OR worry OR "physiological stress" OR angst OR depress* OR mood OR irritab* OR sad* OR affect* OR fatigue OR hope* OR neurotic* OR grief OR griev* OR bereav* OR loss OR burnout OR "burned out" OR "burnt out" OR trauma OR trauma* OR post-traumatic OR post-trauma OR PTSD OR "Post traumatic stress disorder" OR "post-traumatic stress disorder" OR "posttraumatic stress disorder" OR anger OR "substance abuse" OR "substance use" OR "substance use disorder" OR "substance dependence" OR "drug use" OR "drug abuse" OR "drug dependence" ) AND AB ( review OR "systematic review" OR "narrative review" OR "meta-analysis" OR "metaanalysis" OR "meta analysis" OR "literature review" OR synthes* )

Web of Science

(( ALL=(coronavirus OR "corona virus" OR "coronavirus infect*" OR "corona virus infect*" OR "betacoronavirus*" OR "beta coronavirus*" OR "beta corona virus*" OR wuhan OR hubei OR "severe acute respiratory syndrome coronavirus 2" OR "SARS CoV-2" OR "SARSCoV 2" OR "sarscov" OR "cov2" or "sars 2" OR COVID OR "coronavirus 2" OR "covid19" OR "Covid-19" OR "Covid 19" OR nCov OR "new corona virus" OR "new coronavirus" OR "novel coronavirus" OR "novel corona virus" OR ncp OR corona OR pandemic OR "global pandemic" OR epidemic OR "global epidemic" OR coronaviridae) AND ALL=(mental OR psycholog* OR "distress" OR "psychiatr*" OR "social stigma" OR stigma OR fear OR phobia OR anxiety OR anxi* OR stress OR worry OR "physiological stress" OR angst OR depress* OR mood OR irritab* OR sad* OR affect* OR fatigue OR hope* OR neurotic* OR grief OR griev* OR bereav* OR loss OR burnout OR "burned out" OR "burnt out" OR trauma OR trauma* OR post-traumatic OR post-trauma OR PTSD OR "Post traumatic stress disorder" OR "post-traumatic stress disorder" OR "posttraumatic stress disorder" OR anger OR "substance abuse" OR "substance use" OR "substance use disorder" OR "substance dependence" OR "drug use" OR "drug abuse" OR "drug dependence") AND AB=(review OR "systematic review" OR "narrative review" OR "meta-analysis" OR "metaanalysis" OR "meta analysis" OR "literature review" OR synthes*))) |

Cochrane

(coronavirus OR "corona virus" OR "coronavirus infect*" OR "corona virus infect*" OR "betacoronavirus*" OR "beta coronavirus*" OR "beta corona virus*" OR wuhan OR hubei OR "severe acute respiratory syndrome coronavirus 2" OR "SARS CoV-2" OR "SARSCoV 2" OR "SARSCoV2" OR "cov2" or "sars 2" OR COVID OR "coronavirus 2" OR "covid19" OR "Covid-19" OR "Covid 19" OR nCov OR "new corona virus" OR "new coronavirus" OR "novel coronavirus" OR "novel corona virus" OR ncp OR corona OR pandemic OR "global pandemic" OR epidemic OR "global epidemic" OR coronavirinae) AND (mental OR psycholog* OR "distress" OR "psychiatr*" OR "social stigma" OR stigma OR fear OR phobia OR anxiety OR anxi* OR stress OR worry OR "physiological stress" OR angst OR depress* OR mood OR irritab* OR sad* OR affect* OR fatigue OR hope* OR neurotic* OR grief OR griev* OR bereav* OR loss OR burnout OR "burned out" OR "burnt out" OR trauma OR trauma* OR post-traumatic OR post-trauma OR PTSD OR "Post traumatic stress disorder" OR "post-traumatic stress disorder" OR "posttraumatic stress disorder" OR anger OR "substance abuse" OR "substance use" OR "substance use disorder" OR "substance dependence" OR "drug use" OR "drug abuse" OR "drug dependence")

**Suppl figure 1:** AMSTAR 2 ratings of included reviews

| **Study** | **Item 1** | **Item 2** | **Item 3** | **Item 4** | **Item 5** | **Item 6** | **Item 7** | **Item 8** | **Item 9** | **Item 10** | **Item 11** | **Item 12** | **Item 13** | **Item 14** | **Item 15** | **Item 16** | **Rating** |
| --- | --- | --- | --- | --- | --- | --- | --- | --- | --- | --- | --- | --- | --- | --- | --- | --- | --- |
| Systematic reviews with meta-analysis | | | | | | | | | | | | | | | | | |
| Ceban 2021 | Yes | Partial Yes | Yes | Partial Yes | Yes | Yes | No | Partial Yes | Yes | No | Yes | No | Yes | No | Yes | Yes | Low |
| Fond 2021 | Yes | Partial Yes | Yes | Partial Yes | Yes | Yes | No | Partial Yes | Yes | No | Yes | No | No | No | Yes | Yes | Critically low |
| Liu 2021 | Yes | Partial Yes | Yes | Partial Yes | Yes | Yes | Yes | Yes | Yes | Yes | Yes | Yes | Yes | Yes | Yes | Yes | High |
| Toubasi 2021 | Yes | Partial Yes | Yes | Partial Yes | Yes | Yes | No | Partial Yes | Yes | No | Yes | Yes | Yes | Yes | Yes | Yes | Low |
| Vai 2021 | Yes | Partial Yes | Yes | Partial Yes | Yes | Yes | No | Partial Yes | Yes | Yes | Yes | Yes | Yes | Yes | Yes | Yes | Low |
| Narrative reviews | | | | | | | | | | | | | | | | | |
| Fornaro 2021 | Yes | Yes | Yes | Partial Yes | Yes | Yes | Yes | Partial Yes | No | No | NA | NA | No | No | NA | Yes | Low |
| Karaoulanis 2021 | Yes | No | Yes | Partial Yes | No | No | No | Yes | No | No | NA | NA | No | No | NA | No | Critically low |
| Lemieux 2021 | Yes | No | Yes | Partial Yes | Yes | Yes | No | Yes | No | No | NA | NA | No | No | NA | Yes | Critically low |
| Murphy 2021 | Yes | No | Yes | Partial Yes | Yes | Yes | No | Partial Yes | No | No | NA | NA | No | No | NA | Yes | Critically low |

The AMSTAR-2 items refer to 1. PICO research question; 2. Protocol/methods established before review; 3. Selection of study designs; 4. Literature search strategy; 5. Study selection in duplicate; 6. Data extraction in duplicate; 7. List of excluded studies; 8. Description of included studies; 9. RoB; 10. Sources of funding for included studies; 11. Statistical combination of results; 12. Impact of RoB on the results; 13. Account for RoB when interpreting/discussing; 14. Heterogeneity; 15. Publication bias; 16. Conflict of interest. NA = not assessed.

Adaptations to item 9: for ‘partial yes’, the employed tool must have assessed all of the following domains: Selection, Outcome assessment, Analysis. For full ‘yes’, the employed tool must have assessed all of the following domains: Selection, outcome assessment, analysis, selective reporting, conflict of interest
